# Tuberous sclerosis complex: The critical role of the interventional radiologist in management

**DOI:** 10.4102/sajr.v25i1.2034

**Published:** 2021-03-30

**Authors:** Puneet Garg, Anuradha Sharma, Heena Rajani, Apratim R. Choudhary, Rajkumar Meena

**Affiliations:** 1 Department of Radiology, Vardhman Mahavir Medical College and Safdarjung Hospital, New Delhi, India

**Keywords:** tuberous sclerosis complex, angiomyolipoma, selective renal embolisation, hamartomas, ruptured angiomyolipoma, renal haemorrhage

## Abstract

Tuberous sclerosis complex (TSC) is an autosomal dominant neurocutaneous syndrome that is characterised by hamartomas in multiple organs, the characteristic imaging features of which are illustrated in this case report. Angiomyolipoma (AML) is the most common renal manifestation of TSC, which may present with life-threatening haemorrhage at the time of diagnosis. Interventional management with selective renal embolisation is currently the treatment of choice for the safe and effective management of ruptured renal AML.

## Introduction

Tuberous sclerosis complex (TSC) is the second most commonly reported neurocutaneous syndrome following neurofibromatosis type 1, with an incidence of one in 6000.^1^ It follows an autosomal dominant pattern of inheritance and is caused by mutations in two tumour-suppressor genes, TSC1 and TSC2, encoding hamartin and tuberin, respectively. Two-thirds of these mutations may be sporadic.^1,2^ Tuberous sclerosis complex is characterised by hamartomas in various organs, most commonly the skin, brain, eyes, lungs, kidneys, liver, heart and bones with characteristic clinico-radiological manifestations, illustrated herein. The radiologist plays a pivotal role in successful management of a ruptured renal angiomyolipoma (AML), a life-threatening emergency in patients with TSC, which is also highlighted.

## Case presentation

A 24-year-old female presented to the emergency department with complaints of left flank pain and gross haematuria, following a fall. She was conscious and oriented; however, her higher mental functions were subnormal for age. She was experiencing hypovolemic shock and hypotension (blood pressure 90/60 millimetre of mercury [mmHg]) with a feeble pulse and tachycardia (pulse rate of 110/min). Her extremities were cold and clammy. The blood oxygen saturation was normal. She had a diffusely tender swelling over the left lumbar region. Further examination revealed dark papulonodular lesions over her face in a butterfly distribution (adenoma sebaceum), multiple dark macules over her body and focal gigantism of the left thumb. The patient or her relatives did not have any current or prior history of neurological or respiratory complaints.

**FIGURE 1:**
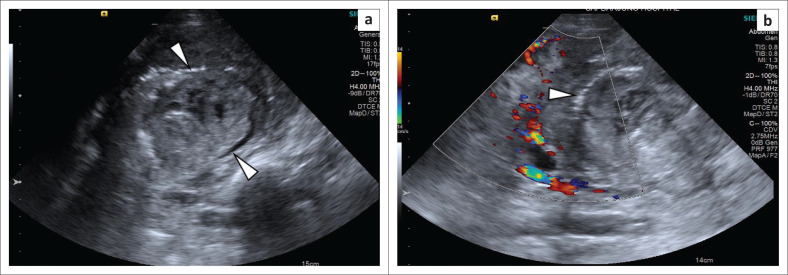
Grey scale (a) and Colour Doppler ultrasound (b) of the left kidney shows a large hetero-echoic left renal mass with intense vascularity at the periphery of the mass and a central avascular component (white arrow heads).

**FIGURE 2:**
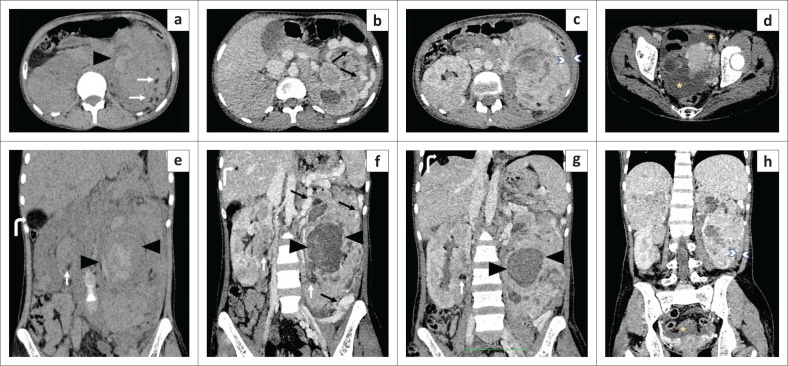
The axial pre-contrast (a) and post-contrast (b-d) and coronal reformatted pre-contrast (e) and post-contrast (f-h) computed tomography (CT) images demonstrating renal and hepatic angiomyolipomas (AMLs). Multiple bilateral renal AMLs have small foci of fat attenuation within (white arrows; a, e). There is heterogeneous enhancement of these renal masses with multiple dilated vascular channels (black arrows) within the left renal mass suggestive of intra-lesional aneurysms and dilated draining venous channels at the periphery. A large hyperdense non-enhancing intra-tumoural haematoma (black arrowheads) is seen within the largest left-sided AML (measuring 17 cm in the largest dimension), which had ruptured. A thin perinephric haematoma (white arrowheads; c, h) was seen on the left. Free fluid was also seen in the pelvis (yellow asterisks; d, h). Well-defined fat attenuated round lesions scattered in the liver suggestive of lipid-rich hepatic AMLs (bent arrow; e-f).

After fluid resuscitation, she was referred for an abdominal ultrasound (US), which revealed massive left renal enlargement with a heterogenous lesion replacing the lower pole and inter-polar region of left kidney. The lesion demonstrated intense internal vascularity and a central avascular hyperechoic area (Figure 1). Multiple heterogeneously hyperechoic lesions were also seen in the right kidney and liver, with propagation velocity artefact causing apparent discontinuity of the right hemidiaphragm immediately superficial to a hepatic lesion. Free fluid with low-level internal echoes was seen in the pelvis. An abdominal contrast-enhanced computed tomography (CECT) scan confirmed a large lesion arising from the mid- and lower pole of the left kidney with an enhancing soft-tissue component, areas of fatty attenuation and bizarre dilated vascular channels. There was a round hyperdense non-enhancing area within the centre of the lesion, corresponding to a large intra-tumoural haematoma. Similar, but smaller lesions were also seen in the right kidney along with fat-containing lesions in the liver (Figure 2). The abdominal findings suggested multiple renal and hepatic AMLs with rupture of the large left renal AML causing a thin perinephric haematoma and associated haemoperitoneum.

**FIGURE 3:**
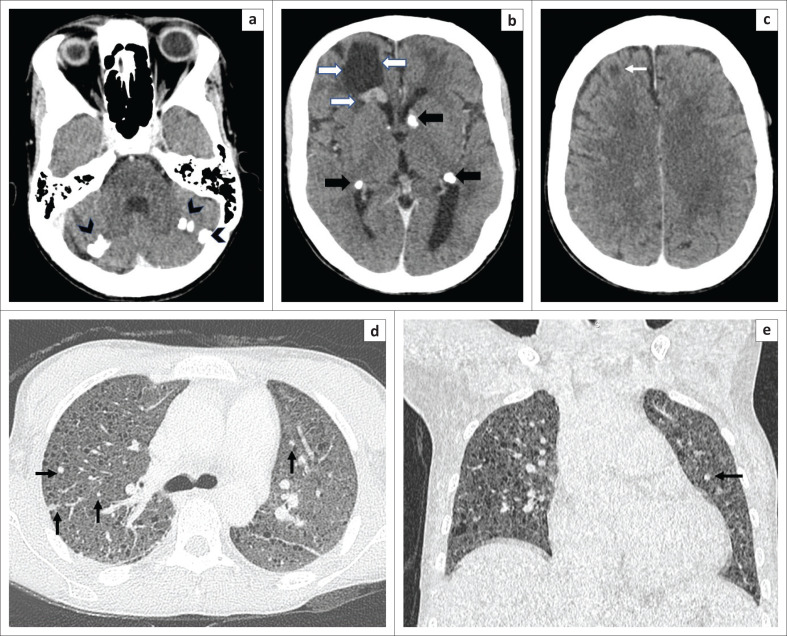
The serial contrast-enhanced computed tomography (CT) brain images demonstrate coarsely calcified cortical tubers in both cerebellar hemispheres (arrowheads, a), multiple calcified subependymal nodules along the walls of both lateral ventricles (thick black arrows, b) with a subependymal giant cell tumour (SEGCT) and associated peri-tumoural cyst in relation to the frontal horn of the right lateral ventricle, extending to the white matter of the right frontal lobe (thick white arrows, b) No hydrocephalus is seen as the tumour is away from the foramen of Monro, the usual location of SEGCT. Multiple hypodense non-calcified cortical tubers were also seen in the cerebral parenchyma (thin white arrow, c). The axial (d) and coronal (e) CT thorax lung windows show multiple small thin-walled cysts scattered diffusely throughout the lung parenchyma without any zonal predilection, suggestive of lymphangioleiomyomatosis (LAM). A few randomly distributed nodules are also seen in both upper lobes (black arrows) representing multifocal micronodular pneumocyte hyperplasia (MMPH).

The study was extended to include computed tomography (CT) imaging of the brain and chest. A CECT scan of the brain revealed multiple cerebral and cerebellar calicified, as well as non-calcified cortical tubers, subependymal calcified nodules and a large cystic lesion with an enhancing mural nodule in the subependymal region of the frontal horn of the right lateral ventricle, consistent with a subependymal giant cell tumour (Figure 3). A CECT of the thorax was performed to evaluate for thoracic manifestations of TSC, revealing multiple, round, thin-walled cysts and a few small randomly distributed nodules uniformly scattered across both lungs, without any pneumothorax or chylous effusions. The findings were consistent with lymphangioleiomyomatosis (LAM) and multifocal micronodular pneumocyte hyperplasia (Figure 3). Bones showed diffuse calvarial thickening and sclerotic foci in the vertebrae, as well as in the pelvis.

**FIGURE 4:**
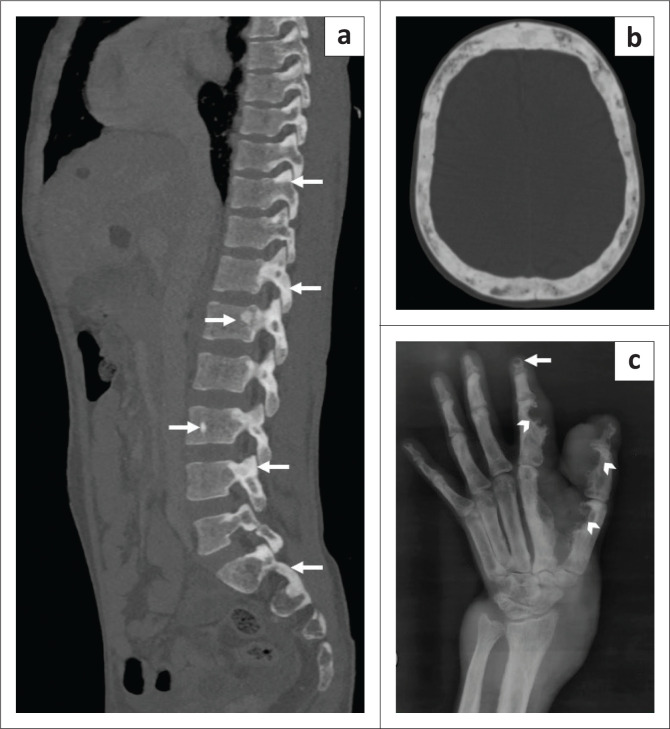
Bone manifestations of tuberous sclerosis complex. Reformatted sagittal computed tomography (CT) in bone window (a) shows patchy and round sclerotic lesions (white arrows) involving the posterior elements and vertebral bodies at multiple levels in the thoracolumbar spine. The axial bone window of the CT head (b) demonstrates a diffusely thickened calvarium with patchy sclerosis. An oblique radiograph of the left hand (c) shows typical intra-osseous cysts in the terminal phalanges (white arrows). The left thumb and second digit (b) additionally show multiple cortical defects or scalloping (arrow heads) and periostitis with associated soft-tissue components, likely representing large cutaneous angiofibromas or hamartomas.

Radiographs of the left hand depicted phalangeal intra-osseous cysts and a large soft-tissue mass causing scalloping of the cortex in the underlying digits (Figure 4).

The dermatological manifestations along with the typical radiological features in the brain, lungs, liver and kidneys helped us to establish the diagnosis of TSC manifesting with an initial presentation of a ruptured renal AML.

## Management and outcome

In view of persistent haematuria, the patient was transferred to the Digital Subtraction Angiography (DSA) suite for emergency endovascular embolisation of the ruptured left renal AML, after obtaining informed consent. The pre-procedural investigations revealed a reduced haemoglobin level of 4.1 grams per decilitre (g/dL), normal platelet count (1 79 000/mm^3^), total leukocyte count (8400/mm^3^) and kidney function parameters (blood urea: 28 g/dL, serum creatinine 0.7 mg/dL). The coagulation profile was normal (prothrombin time: 12 s and International normalised ratio [INR]: 1.1).

### Selective arterial embolisation procedure

**FIGURE 5:**
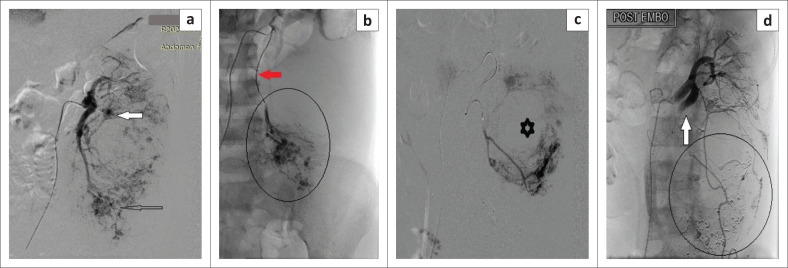
Digital subtraction angiography images of the selective renal embolisation procedure: (a) catheter angiogram of the left main renal artery shows a solitary aneurysm (solid white arrow) and multiple clustered aneurysms in the lower pole (open arrow), suggestive of abnormal intra-tumoural vascularity in a renal angiomyolipoma. (b) Super-selective angiogram using a microcatheter (red arrow) indicates tumoural hypervascularity and clustered small aneurysms in the lower pole of the kidney (encircled). (c) Super-selective angiogram reveals the rounded area of absent parenchymal blush (star) suggestive of haematoma. There is hypervascularity in the surrounding parenchyma. (d) Post-embolisation catheter angiogram from the main renal artery demonstrated the de-vascularised arterial stump (white arrow). Embolised tumoural vessels with glue cast and alcohol (encircled). The upper pole of the kidney is spared and shows normal enhancement.

Under sterile conditions and local anaesthesia, right common femoral arterial access was secured using a 6 French (Fr) sheath. A 5Fr-renal double curve catheter (Cook Medical, Bloomington, USA) was used to obtain an angiogram of the left main renal artery which revealed massive enlargement of the renal outline, hypertrophy of the dorsal branch of the renal artery supplying the AML, multiple aneurysms at the lower pole and an associated bizarre pattern of tumoural hypervascularity (Figure 5a).

Delayed frames indicated dilated venous channels along the margins of the kidney. The upper pole indicated preserved renal parenchymal vascularity with multiple small areas of tumoural blush. No accessory renal arteries were identified and no suprarenal or lumbar accessory supply to the lesion was demonstrated. Using a 2.7Fr-microcatheter, super-selective angiograms were obtained from multiple segmental branches of the dorsal branch of left renal artery, which revealed tumoural hypervascularity, multiple clustered aneurysms (Figure 5b) and a hypo-vascular region in the centre of the renal outline, splaying the vasculature and corresponding to the intra-tumoural hematoma (Figure 5c). A few of these branches were embolised using 10 mL absolute alcohol mixed with lipiodol^®^ (Guerbet, Aulnay-sous-Bois, France) at a ratio of 4:1 and poly-vinyl alcohol (PVA) particles of size 500 µm –700 µm, and some were embolised with 0.6 mL 25% N-butyl-cyanoacrylate (NBCA) mixture with lipiodol^®^ at a ratio of 1:3. The post-embolisation angiogram revealed complete occlusion of the abnormal tumoural vessels with preservation of the normal renal parenchyma (Figure 5d). Local anaesthetic and intravenous analgesics were administered to the patient during the procedure.

In the immediate post-procedure period, the patient’s blood pressure was 110/70 mm of Hg with a pulse rate of 100 per minute. She received three units of packed red blood cells. On follow-up (after 72 h), the haematuria had resolved with stabilisation of the vitals and haemoglobin levels (8.2 g/dL). The kidney function tests remained normal (blood urea: 28 mg/dL, serum creatinine 0.8 mg/dL). She was referred to the Department of Respiratory Medicine and Neurosurgery for management of the pulmonary and central nervous system manifestations. A follow-up CT was planned for 3 months post-procedure.

### Ethical considerations

This article followed all ethical standards for research.

## Discussion

**FIGURE 6:**
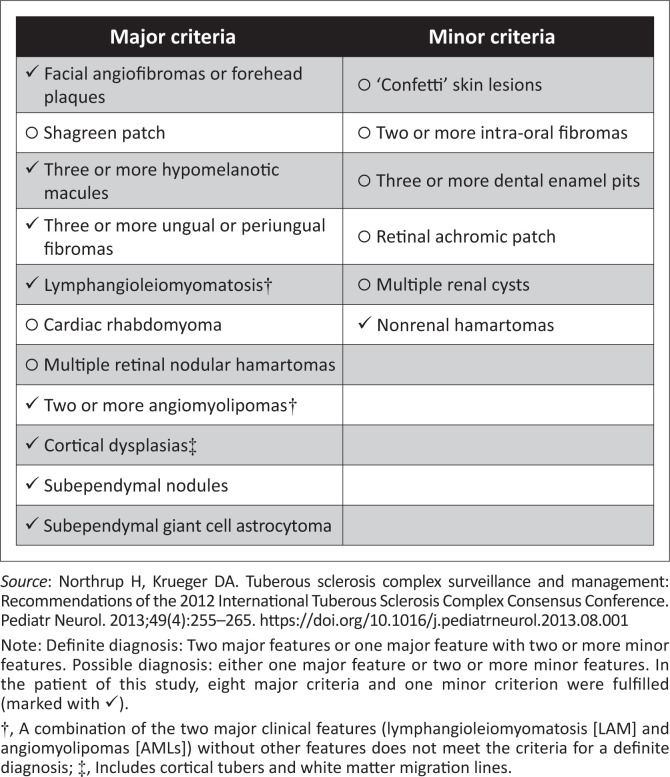
Summary of major and minor criteria for the diagnosis of tuberous sclerosis.

Tuberous sclerosis complex is a syndrome with multisystem involvement. The classic Vogt’s triad of seizures, mental retardation and adenoma sebaceum is found only in 30% – 40% of patients with TSC.^1^ The *2012 International Tuberous Sclerosis Complex diagnostic criteria* are summarised in Figure 6.^3^ The patient fulfilled eight major criteria and one minor criterion, indicating a ‘definite’ diagnosis of TSC with the classic imaging features described above. Imaging plays a crucial role not only in the diagnosis but also in the follow-up of patients with TSC. The guidelines for imaging surveillance in TSC are summarised in Table 1.^4^

**TABLE 1:** Guidelines for imaging surveillance of tuberous sclerosis complex.

Organ	Newly diagnosed or suspected case of TSC	Already diagnosed with definite or probable TSC
Brain	MRI brain for all patients	MRI of the brain every 1–3 years in asymptomatic TSC patients <25 years age, without SEGA. MRI of the brain every 1–3 years in asymptomatic TSC patients, with SEGA; more often if SEGA is growing in size.
Kidney	MRI of the abdomen If MRI is contraindicated, perform CT or US scan	Patients with renal lesions should perform annual imaging of the abdomen for assessing the progression of AML, renal cystic disease and occurrence of the rare renal cancer throughout their lifetime. In the absence of renal lesions, the scans should be repeated every 1–3 years through childhood and early adult life: ■ MRI is the preferred modality. US or CT scan to be considered if it is not feasible to perform MRI annually (due to limited availability or need of general anaesthesia) and if on MRI the anatomy and pathology are judged to be easy to interpret by US.
Lung	Baseline high-resolution computed tomography (HRCT) Thorax in females of child-bearing age and symptomatic adult males	If there is no evidence of LAM on baseline HRCT, obtain HRCT every 5–10 years in asymptomatic females of child-bearing age until menopause. If LAM detected on HRCT on baseline HRCT, follow-up with pulmonary function testing.
Heart	Baseline echocardiogram in all children and symptomatic adults.	Echocardiogram should be repeated every 1–3 years in asymptomatic patients until either complete regression of cardiac rhabdomyomas or until the first sign of regression.

*Source*: Adapted from Amin S, Kingswood J, Bolton P, et al. The UK guidelines for management and surveillance of Tuberous Sclerosis Complex. QJM. 2018;112(3):171–182. https://doi.org/10.1093/qjmed/hcy215

HRCT, high-resolution computed tomography; MRI, magnetic resonance imaging; TSC, tuberous sclerosis complex; SEGA, Subependymal giant cell astrocytoma; US, ultrasound scan; CT, computed tomography; LAM, lymphangioleiomyomatosis; AML, angiomyolipoma.

Renal AML is the most common benign renal tumour with a prevalence rate of about 0.3% – 3% worldwide.^5^ Approximately 20% of patients with renal AML are diagnosed with TSC, and conversely, 55% – 75% of patients with TSC have renal AMLs.^2^ As the nomenclature suggests, AML is composed of dysmorphic, tortuous vascular tissue with smooth muscle and fat components. Sporadic cases are generally unilateral, whereas those associated with TSC are multiple, bilateral and more aggressive.^6^ Blood vessels in AML are fragile as they lack internal elastic lamina, thus forming multiple aneurysms. Although most renal AMLs are usually asymptomatic, patients with enlarged kidneys and AML are more susceptible to trauma and may present with spontaneous perinephric haematoma, a palpable flank mass with pain or haematuria.^2,5,6^ It has been reported that AMLs associated with TSC are more prone to rupture than sporadic AMLs.^7,8^ The following are the predictors of the risk of haemorrhage in AML: size >4 centimetres (cm) (in sporadic AML) and >3.5 cm in AML associated with TSC, intra-tumoural aneurysms measuring >5 mm, female sex, pregnancy and increased oestrogen levels.^9,10^

Renal artery embolisation is established as the treatment of choice for a ruptured AML.^10^ The first case was described in the literature in 1984.^11^ Embolisation may also be used as a prophylactic procedure in AML to prevent rupture or bleeding and preoperatively to reduce intraoperative bleeding.^12^ The widely accepted criteria for prophylactic nephron sparing treatment of asymptomatic AML is a size of >4 cm or other risk factors of tumoural rupture listed earlier, especially in female patients of child-bearing age and those with limited access to follow-up and emergency care.^6,13^ Although historically, surgery was the accepted treatment of choice, the role of endovascular management has emerged as it is effective, has a low complication rate, preserves renal function and has ease of repeatability.^14^ However, if there is suspicion of malignancy in the renal mass or if the patient is pregnant, surgery is the treatment of choice.^6,15^ The goal of endovascular management in AMLs is to obliterate the tumoural capillaries and small arterioles harbouring the small aneurysms. Surgical options like nephron sparing surgery carry the risk of increased morbidity, especially when lesions are multiple, as in TSC.^9^ Super-selective embolisation is safe and highly effective, especially in those who harbour multiple AMLs in both kidneys. Other interventional ablative procedures available involve radiofrequency ablation, microwave ablation and cryoablation.^6^ Skilled super-selective embolisation procedures cause minimal collateral damage to the normal renal parenchyma. Moreover, these minimally invasive procedures can be repeated in the case of recurrence or technical failure on an initial attempt. These procedures are carried out under local anaesthesia circumventing the risk of general anaesthesia, particularly in patients with TSC who may have pre-existing respiratory and hepatic compromise.

Classically, absolute alcohol with PVA particles is the embolic agent of choice, which helps in achieving permanent devascularisation of the lesion with subsequent reduction in size and haemorrhage risk. Alcohol causes permanent occlusion at the level of the arterioles and capillary bed by triggering perivascular necrosis and intravascular thrombosis.^13^ Many studies have documented reduction in the size of AML after embolisation, ranging from 20% to 70%.^14,15,16,17^ Balloon occlusion catheters are often used with absolute alcohol because it occludes the vessel proximally, thereby decreasing the blood flow and increasing the contact time of alcohol with the affected blood vessels. Balloon occlusion also prevents reflux of ethanol, thereby avoiding non-target embolisation.^18^ N-butyl-cyanoacrylate or glue is a permanent liquid embolic agent, which is diluted in iodised oil, lipiodol. It flows into the tumoural vessels and aneurysms, forming a solid glue cast as it comes into contact with blood.^17^ The speed of polymerisation of the glue depends on the concentration of NBCA in lipiodol. Varying proportions (1:3 to 1:6) have been used to achieve safe and adequate embolisation.^17^ We used NBCA with lipiodol) for embolisation at a 1:3 ratio in the patient to de-vascularise part of the tumour, in addition to alcohol and PVA. A wide variety of embolic agents have been used for embolising renal AML (Table 2).^12,16,17^ However, no study has revealed superiority of one over another.^18^

**TABLE 2:** Embolic agents used for embolisation of angiomyolipoma by various authors in the past.

Author	Number of lesions	Embolic agents used
Rimon et al.^12^	48	Alcohol plus PVA
Lee et al.^16^	11	Gelfoam and coils
Bardin et al.^17^	23	PVA, coils and glue

AML, angiomyolipoma; PVA, poly-vinyl alcohol.

Coils should be avoided in AML embolisation as they cause proximal occlusion, promoting formation of collaterals distal to the level of occlusion, thereby increasing the risk of recurrence. Furthermore, using coils precludes a re-embolisation procedure by establishing loss of arterial access to the tumoural feeders.

The short-term complications of embolisation include post-embolisation syndrome manifesting as pain and fever, which can be managed with analgesics. Non-target embolisation is common with glue and alcohol and may cause renal parenchymal damage leading to hypertension and decreased renal function. Renal abscess and tumoural rupture are other complications of embolisation. Access-site complications such as groin haematoma and pseudoaneurysm may also occur, as observed with other catheter-based angiographic procedures.^18^ The main long-term complication includes recurrence of the lesion with a recurrence rate varying between 11% and 40%.^18^ ‘Fat-poor’ AMLs respond better to endovascular management because of the larger proportion of angiomyogenic component with a lower rate of recurrence.^13,17^

## Conclusion

The radiologist plays a central role not just in the diagnosis of TSC and detection of its multisystem involvement but also in the interventional management of renal AML, which may present with life-threatening haemorrhage. Selective endovascular embolisation can be used to de-vascularise the lesion and control the bleeding, thus proving to be a safe and effective treatment option in such patients.
